# Ultrasound-Assisted Processing of Aluminum Matrix Nanocomposites: Parameter Optimization for Enhanced Mechanical Properties

**DOI:** 10.3390/ma19091876

**Published:** 2026-05-02

**Authors:** Yesufikad Fentie Takele, Abraham Debebe Woldeyohannes, Saša Milojević, Slavica Miladinović, Mladen Radojković, Blaža Stojanović

**Affiliations:** 1 Department of Mechanical Engineering, Nano Material Center of Excellence, Addis Ababa Science and Technology University, Addis Ababa P.O. Box 16417, Ethiopia; abraham.debebe@aastu.edu.et; 2 Faculty of Engineering, University of Kragujevac, Sestre Janjić 6, 34000 Kragujevac, Serbia; slavicam@kg.ac.rs (S.M.); blaza@kg.ac.rs (B.S.); 3 Faculty of Technical Sciences, University of Priština, Knjaza Miloša 7, 38220 Kosovska Mitrovica, Serbia; mladen.radojkovic@pr.ac.rs

**Keywords:** Al_2_O_3_ nanoparticle, ultrasonication, Taguchi optimization, mechanical properties, microstructural analysis

## Abstract

This study investigates the enhancement of AA6082/Al_2_O_3_ aluminum metal matrix nanocomposites (AMMNCs) through powder metallurgy combined with systematic process optimization. Ultrasound-assisted dispersion and Taguchi design L9 orthogonal array were employed to improve nanoparticle distribution and optimize fabrication parameters. The effect of Al_2_O_3_ content and ultrasonication time (UT) on hardness and compressive strength was analyzed using S/N ratio and ANOVA. Characterization was performed using X-ray Diffraction (XRD) and scanning electron microscopy (SEM). The result shows that Al_2_O_3_ content had the most significant influence on both hardness (82.25%) and compressive strength (81.08%), followed by UT. The optimal condition produced a maximum hardness of 31.9 HV and compressive strength of 205.53 MPa. Regression models demonstrated strong predictive accuracy (R^2^ > 85%). Overall, the study highlights the effectiveness of parameter optimization in improving nanocomposite performance and provides valuable guidance for advanced material design.

## 1. Introduction

Composite materials are increasingly preferred over conventional metals due to their lightweight, high strength and stiffness, design flexibility, and superior resistance to corrosion and cracking [1]. Metal matrix composites (MMCs), in particular, are gradually replacing traditional materials because of their excellent properties and the growing demand for lightweight, high-strength engineering solutions [2]. This shift also improves efficiency and reduces costs, especially in automotive and aerospace applications [3].

The demand for advanced materials has further promoted the development of ceramic particle–reinforced MMCs [4], which offer enhanced strength, thermal stability, and tribological performance compared to conventional alloys [5]. Among these MMCs, aluminum metal matrix composites (AMMCs) have gained significant attention, due to their low density, high strength, and excellent wear resistance [6]. The high specific strength, wear resistance, and fatigue durability of AMMCs make them widely used in structural applications [7].

Driven by the demand for lightweight materials, aluminum alloys have become a preferred alternative to heavier metals such as steel and copper [8,9], due to their good mechanical properties, recyclability, and environmental benefits [10]. Aluminum alloys are widely used in transportation [11], aerospace [12], structural engineering [13], and marine applications [14] because of their low density, affordability, ductility, and corrosion resistance. However, limitations such as relatively low strength, high wear rate, and poor thermal stability restrict their broader applications [15,16]. Consequently, the search for lightweight, high-performance materials has accelerated the transition from conventional alloys to advanced composite materials in recent decades [17].

The performance of MMCs can be significantly improved by adding suitable reinforcements. In recent years, ceramics particles such as SiC and Al_2_O_3_ have been widely used [18], along with B_4_C, TiC [19], ZrO_2_, Tib_2_, AlN [20], graphite, MoS2, and ZrB2. These reinforcements enhance composite properties by combining the inherent properties of metals with the unique properties of ceramic particles, leading to improved mechanical, thermal, and tribological performance of MMCs [21,22,23]. Compared with conventional MMCs, nanoparticle-reinforced composites exhibit superior mechanical performance due to their unique interfacial bonding and uniform dispersion [24,25,26]. Even a small amount of well-dispersed nanoparticles (≈1%) can substantially strengthen aluminum or magnesium matrices [27]. Moreover, nanoparticle reinforcement enhances wear resistance, damping capacity, and mechanical strength of MMCs, expanding their application [28]. Incorporating nanoparticles into aluminum matrices enables tailored material properties, significantly improving mechanical strength, wear resistance, and thermal conductivity, which makes these nanocomposites highly attractive for a wide range of engineering applications [29]. Consequently, aluminum-based nanocomposites have gained widespread use in aerospace, automotive, and marine industries, driven by their high strength-to-weight ratio, improved wear and corrosion resistance, and potential for lightweight, energy-efficient components [30,31].

Although nanoparticle reinforcement significantly improves nanocomposite performance, achieving uniform nanoparticle dispersion is crucial for ensuring the quality of metal matrix nanocomposites (MMNCs) [32]. A homogeneous distribution of nano-sized ceramic particles is therefore essential for producing high-quality MMNCs [33]. Metal matrix composites are fabricated using various processes [34]; among these, stir casting is generally acknowledged as a commercially viable and promising technique, offering advantages in simplicity, flexibility, and cost-effective high-volume production. However, attaining homogeneous particulate distribution within the matrix remains a substantial challenge [35]. Consequently, a major limitation of stir casting, specifically reinforcement segregation, necessitates the use of powder metallurgy (PM) for AMMC fabrication [36]. PM offers a viable solution by facilitating uniform particle distribution [20,37]. Achieving consistent and even dispersion of micro/nano-reinforcements within the matrix material remains challenging. A PM approach that uses high-energy milling is one possible way to overcome this challenge. However, problems like agglomeration and clustering in bulk materials can still exist [38].

The performance of MMNCs is governed by both the fabrication technique and nanoparticle distribution uniformity. While diverse synthesis exists from solid state mixing to powder metallurgy, achieving homogeneous nanoparticle dispersion remains a substantial challenge. Ultrasonic cavitation has emerged as a promising approach, enabling near-uniform nanoparticle distribution in the metal matrix [39]. Ultrasonic dispersion method (UDM) is vital for nanomaterial preparation, which efficiently delivers high ultrasonic energy to minimal volumes to achieve near-complete dispersion of agglomerates [40].

Numerous studies have employed UDM to fabricate nanocomposites. For instance, Simoes et al. [41] explored the production of aluminum and nickel matrix composites reinforced with carbon nanotube (CNT) using powder metallurgy, where ultrasonication was implemented as the dispersion and mixing methodology. Similarly, Rocha and Simoes [29] investigated the fabrication and characterization of Al_2_O_3_ nanoparticle-reinforced AMMNC. They utilized powder metallurgy, incorporating ultrasonication for dispersion and mixing, followed by cold pressing and sintering, to evaluate the microstructure and mechanical properties. Kumar et al. [42] developed a novel solution mixing process to overcome the limitation of standard ball-milling in fabricating uniformly dispersed carbon nanotube reinforced composites without CNT damage. They synthesized Al-0.5 wt.% CNT composites using both ball milling and solution mixing, comparing CNT dispersion, structural, and thermal properties. The solution mixing proved simpler and more effective for achieving uniform CNT distribution without structural damage. Further optimizing dispersion, Shuhari et al. [43] conducted a study utilizing ultrasonic treatment to reduce agglomeration of an Al_2_O_3_/multi-walled carbon nanotube (MWCNT) mixture. They investigated the impact of adding MWCNT to Al_2_O_3_ ceramic composite and varying ultrasonic duration on their dispersion, maintaining a constant 0.5 wt.% MWCNT composition. The mixtures were dried at 80 °C for 12 h before compaction. The study revealed that increased ultrasonic duration improved MWCNT dispersion and minimized agglomeration in the Al_2_O_3_ matrix.

Various optimization techniques are widely used to improve the performance of composite materials. Among them, the Taguchi method is a robust experimental design approach that establishes clear relationships between input parameters and output responses, delivering reliable results with fewer experiments and reduced variability [44]. The Taguchi method identifies optimal process conditions using the signal-to-noise (S/N) ratio, which reflects the balance between desired performance and unwanted variations. The S/N ratio is calculated for each level of the process parameters. In general, a higher S/N ratio indicates better performance, regardless of the type of performance characteristic. Consequently, the optimal process parameter level is identified as the one with the highest S/N ratio [45].

Despite the widespread use of ultrasonic dispersion in nanocomposite fabrication, its application to AA6082/Al_2_O_3_ nanocomposites remains unexplored in current literature. In particular, the individual and combined effects of sonication time and reinforcement concentration on mechanical performance have not yet been systematically investigated. Addressing this gap is important because AA6082 is one of the highest-strength alloys in the 6xxx series (Al-Mg-Si) [46,47,48]. It combines excellent formability, corrosion resistance, and machinability with lightweight and durability, making it ideal for transportation and structural applications. Its mechanical strength is further enhanced through precipitation hardening, a heat treatment process enabled by its primary alloying elements, magnesium (Mg) and silicon (Si) [49,50,51]. Therefore, the main contribution of this work is the development of a novel AA6082/Al_2_O_3_ aluminum matrix nanocomposite fabricated using ultrasonic dispersion combined with Taguchi optimization. Unlike previous studies that primarily focused on reinforcement content or ultrasonication time alone, the present study systematically examines the combined influence of reinforcement content, ultrasonic dispersion, and processing optimization on the mechanical performance of the nanocomposites. The results show improved hardness and compressive strength, and also provide useful guidelines for selecting optimal processing parameters.

## 2. Materials and Methods

The materials utilized in this study include AA6082 as the matrix and Al_2_O_3_ nanoparticles as reinforcement, synthesized via the ultrasonication dispersion method.

### 2.1. Materials

Among aluminum alloys, the 6xxx series (Al-Mg-Si) is notable for its precipitation hardening capability, which enhances strength through heat treatment [52,53]. These alloys offer an exceptional combination of a high strength-to-density ratio, good corrosion resistance, affordability, ease of production, excellent formability and weldability, and high thermal and electrical conductivity [54,55,56]. These versatile properties make them essential in sectors from civilian automobiles to defense applications like military vehicles, rockets, and missiles [57]. The AA6082 alloy currently offers the best combination of properties among the 6xxx series alloys [58]. This study focuses on fabricating aluminum matrix nanocomposites using an AA6082 alloy matrix (composition shown in Table 1) reinforced with aluminum oxide (Al_2_O_3_) nanoparticles. The average particle size of the AA6082 powder is approximately 0.2267 µm, while the average particle size of Al_2_O_3_ nanoparticles was approximately 57 nm, as estimated from XRD analysis. Al_2_O_3_ was chosen as the reinforcement due to its outstanding hardness, thermal stability, and excellent wear and corrosion resistance [59]. Al_2_O_3_ nanoparticles powder (99.9% purity) was purchased from Intelligent Materials Pvt. Ltd., Village Sundran, Derabassi (PB)-140507, India.

### 2.2. Fabrication of Aluminum Matrix Nanocomposites via Ultrasonic Dispersion Method

**Figure 1:**
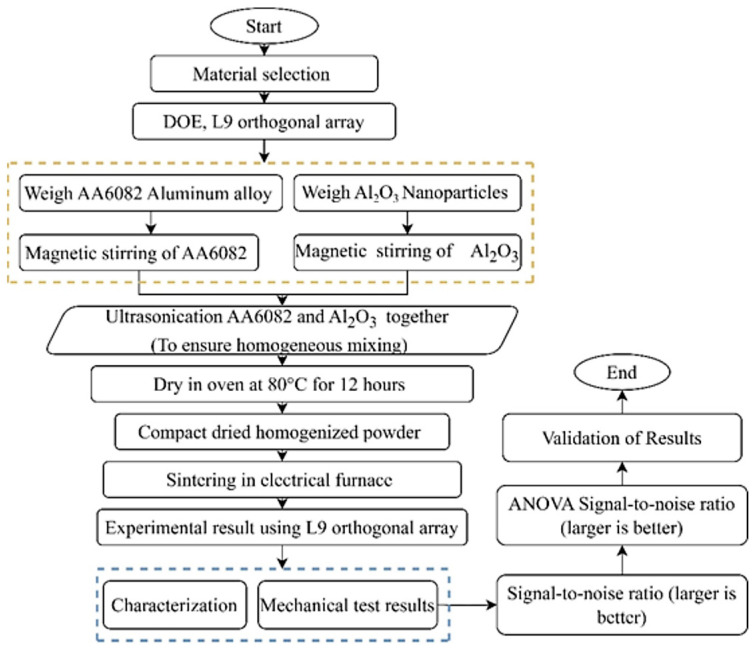
Flow chart of nanocomposites preparation.

The synthesis of nanocomposites through the ultrasonication dispersion method is systematically illustrated in Figure 1, which presents each step of the process, from the initial weighing of AA6082 aluminum alloy and Al_2_O_3_ nanoparticles to the final evaluation of their mechanical and microstructural properties. To ensure a consistent dispersion, the weighted AA6082 aluminum alloy and Al_2_O_3_ nanoparticles were initially stirred separately with a magnetic stirrer. The AA6082 powder was then dispersed in 97% pure ethanol solvent [60], which served as the dispersion medium during ultrasonic blending to promote relatively uniform distribution of Al_2_O_3_ nanoparticles and reduce agglomeration. The low viscosity and surface tension of ethanol improve powder wetting and enhance ultrasonic energy transmission, while also preventing cold welding of aluminum particles. Additionally, its high volatility enables easy evaporation after mixing. Subsequently, the Al_2_O_3_ nanoparticles were gradually added to the suspension and further sonicated using a BANDELIN/SONOPULS ultrasonic homogenizer (BANDELIN electronic, Berlin, Germany), with the total ultrasonic energy input during processing being approximately 135.84 kJ, 207 kJ, and 279 kJ for different ultrasonication durations. To break up nanoparticle agglomerates and achieve a uniform suspension, the treatment was performed at 75 W (Watts) power with an MS73 probe at room temperature, with a 5-s on/3-s off [61]. During the ultrasonication process, aluminum alloy powder and the corresponding Al_2_O_3_ reinforcement were mixed with 150 mL of ethanol solvent in a beaker. The ultrasonic probe was immersed below the solvent surface, and the mixture was subjected to ultrasonic dispersion for a specified duration under controlled power conditions. In this study, nine nanocomposite samples, designated US1 to US9, were prepared, containing 1, 2, and 3 wt.% Al_2_O_3_ nanoparticles. Each sample was subjected to ultrasonic dispersion for 2, 3, and 4 h, respectively, to systematically investigate the effects of ultrasonication duration and nanoparticle concentration on the composite’s mechanical and microstructural properties. After ultrasonication, the mixed powders were filtered and dried in an oven at 80 °C for 12 h in air to ensure complete evaporation of ethanol [62]. The dried homogenized powder was compacted at room temperature under a pressure of 100 MPa using uniaxial cold compaction. The load was applied at a constant rate with a retention time of 5 min, producing cylindrical samples. The compacted samples were sintered in an electrical furnace (30–3000 °C, Nabertherm GmbH, Lilienthal, Germany) at 600 °C for 3 h under vacuum. The sintering temperature was selected to promote diffusion and densification while minimizing excessive oxidation. However, oxidation control and relative density after sintering were not within the scope of the present study. The temperature was gradually increased, and the heating and cooling rates were maintained at 10 °C per minute. Finally, the mechanical properties of sintered samples were evaluated through hardness and compressive strength tests. Additionally, microstructural characterization of the AA6082/Al_2_O_3_ nanocomposites was performed using scanning electron microscopy (SEM) and X-ray Diffraction (XRD) analysis.

### 2.3. Mechanical Properties and Characterization

The mechanical properties and characterization of materials play a pivotal part in determining the performance and behavior of nanocomposites. The fabricated aluminum-based nanocomposites undergo rigorous evaluation through XRD and SEM analyses. Additionally, properties of the nanocomposites, such as hardness and compressive strength, were assessed through Vickers hardness and compressive strength tests. This comprehensive analysis aids in determining the performance capabilities of fabricated nanocomposite materials.

#### 2.3.1. Characterization

To characterize, XRD and SEM analyses were employed. XRD was used to measure crystalline size and material composition. SEM was used to assess the uniformity of Al_2_O_3_ nanoparticle dispersion within the AA6082 matrix.

#### 2.3.2. Mechanical Properties

The mechanical performance of the fabricated AMMNC samples was assessed through hardness and compressive strength tests. Vickers hardness measurements were conducted on the polished aluminum alloy and nanocomposite samples using a digital Vickers hardness tester (Model: HVS-50), SAM WON ENGINEERING CO., Gyeonggi-do, Republic of Korea to determine their resistance to permanent deformation with three repetitions for each sample. The tests were performed in accordance with ASTM E92 [63]. For each sample, three indentations were made, and the average hardness value was reported to ensure measurement reliability under a 5 kgf load with a 15-s dwell time. The compressive strength of both the AA6082 alloy and its nanocomposites was evaluated using a universal testing machine (UTM) manufactured by Jinan Testing Equipment IE Corporation, Jinan, China. Compression testing was carried out in accordance with ASTM E9 standards [64], with three repetitions for each sample to ensure precision and consistency in the measurements. The test specimens were meticulously prepared following these standards to accurately assess the true compressive behavior and mechanical performance of the materials.

### 2.4. Design of Experiment (DOE)

The experimental design was formulated using the Taguchi method to efficiently determine the optimal number of experimental runs and parameter combinations. This approach was chosen for the study due to its ability to achieve optimization with a limited number of experiments [65]. Two key factors were considered: the volume fraction of Al_2_O_3_ nanoparticle and the ultrasonication time, each evaluated at three levels. Based on these parameters and their levels, a Taguchi L9 orthogonal array was developed to investigate the mechanical properties of the aluminum matrix nanocomposites. The design included two factors and nine experimental runs, with each experiment performed three times to ensure accuracy. The factors and their corresponding levels are presented in Table 2, while the detailed L9 experimental design is shown in Table 3.

## 3. Results and Discussion

This section presents and discusses the experimental findings regarding the microstructural characteristics and mechanical properties of the ultrasonicated AA6082/Al_2_O_3_ nanocomposites. It aims to provide a comprehensive understanding of how specific processing parameters, namely ultrasonic time (UT) and Al_2_O_3_ concentration, collectively influence the resulting microstructure and mechanical performance of these AA6082/Al_2_O_3_ nanocomposites.

### 3.1. SEM Analysis of AA6082/Al_2_O_3_ Nanocomposites

**Figure 2:**
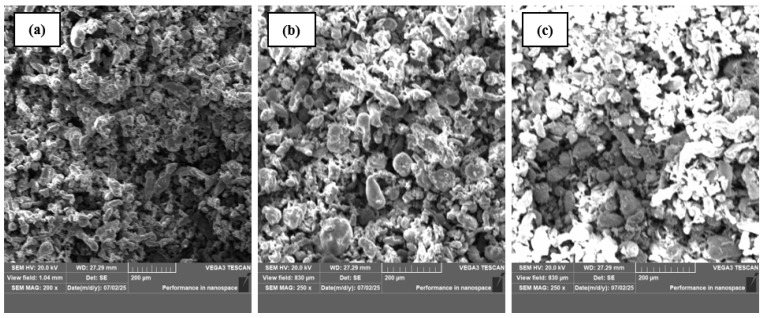
SEM image of AA6082-based nanocomposites. (**a**) AA6082/1 wt.%Al_2_O_3_, (**b**) AA6082/2 wt.%Al_2_O_3_, and (**c**) AA6082/3 wt.%Al_2_O_3_ nanocomposites.

To examine the microstructural behavior of the fabricated AA6082/Al_2_O_3_ aluminum matrix nanocomposites (AMMNCs), the blended powders obtained after compaction were directly used for microstructural characterization in cylindrical form. SEM was employed using a TESCAN VEGA3 SBH system (Product No. 120-0069). The SEM images of the synthesized composites are presented in Figure 2. At lower reinforcement levels, specifically at (a) and (b) (Figure 2), the SEM micrographs reveal relatively uniform dispersion of Al_2_O_3_ throughout the aluminum matrix. This suggests that ultrasonication was effective in breaking up particle clusters and promoting the uniform distribution of the reinforcement at these lower concentrations. However, at a higher reinforcement level of 3 wt.% Al_2_O_3_ (Figure 2c), the microstructure begins to show signs of non-uniform distribution and agglomeration. These observations highlight the importance of optimizing the reinforcement content to achieve a homogeneous microstructure. Maintaining a balance between particle dispersion and loading is crucial for enhancing the overall performance of the composite material.

### 3.2. XRD Analysis of AA6082/Al_2_O_3_ Nanocomposites

**Figure 3:**
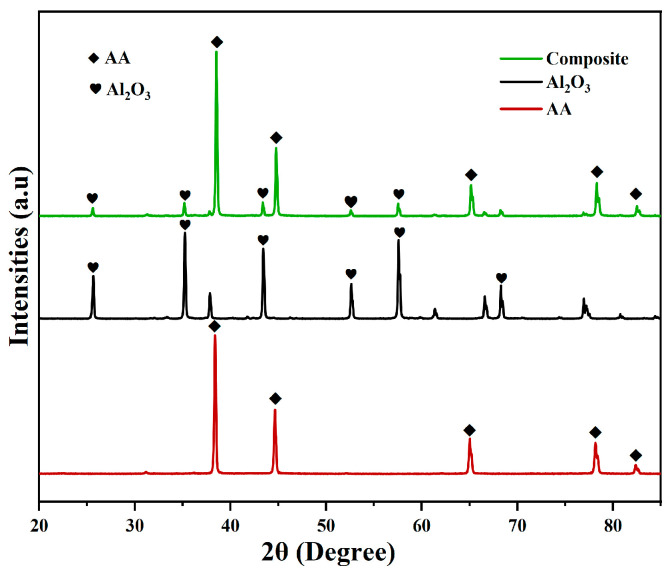
XRD pattern of AA, Al_2_O_3_, and nanocomposite.

The XRD pattern of aluminum alloy (AA), Al_2_O_3_, and nanoparticle reinforced nanocomposite is shown in Figure 3. The particle size of the materials has been determined from XRD peaks, employing Scherrer’s equation: $D=\frac{k\lambda }{\beta cos(\theta )}$, where D is crystallite (particle) size, K is Scherrer constant, λ is the X-ray wavelength (Cu kα), β is the full width at half maximum (FWHM) of the most intense diffraction peak, and θ is the Bragg’s angle in degrees. Based on Scherer’s equation, the average particle size of the Al_2_O_3_ nanoparticles was approximately 57 nm. For XRD analysis, the synthesized composites were prepared in powder form, which were then mounted on a sample holder for analysis. Figure 3 displays the XRD patterns of AA, Al_2_O_3_ nanoparticles, and AA/Al_2_O_3_ nanocomposite. The XRD report clearly confirms the successful incorporation and presence of Al_2_O_3_ nanoparticles within the aluminum metal matrix. Specifically, high intensity peaks for the aluminum alloy were observed at 2θ values of 38.38 and 44.66 degrees. Correspondingly, strong peaks for Al_2_O_3_ were identified at 2θ values of 35.24, 43.42, and 57.58 degrees.

### 3.3. Vickers Hardness of AA6082/Al_2_O_3_ Naocomposites

This section presents the Vickers hardness (HV) results for ultrasonicated AA6082/Al_2_O_3_ aluminum matrix nanocomposites (AMMNCs). Made by mixing AA6082 powder with Al_2_O_3_ nanoparticles in an ethanol medium, the measured hardness reflects the properties of the compacted and sintered material. The study investigates the effect of UT and reinforcement content on the final hardness of the material. The unreinforced AA6082 alloy was used as a control sample, showing a hardness of 30 HV, consistent with the value reported in [66]. This baseline is crucial for evaluating the effects of Al_2_O_3_ nanoparticle incorporation and subsequent processing.

#### Effect of Al_2_O_3_ Addition and Ultrasonication Time on Hardness

**Figure 4:**
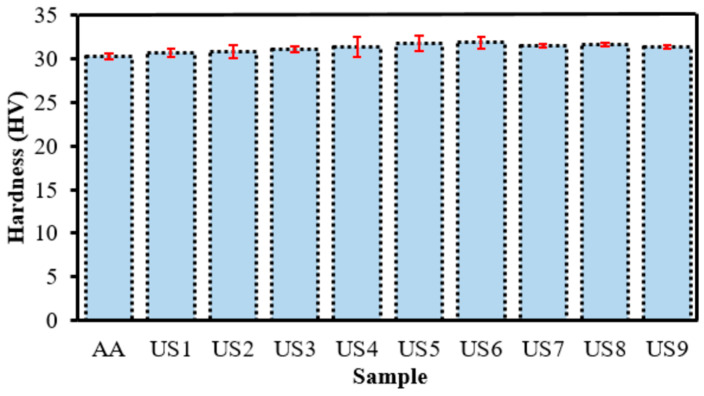
Hardness of nanocomposites.

The results clearly demonstrate that the incorporation of Al_2_O_3_ nanoparticles enhances the hardness of the nanocomposites, as shown in Figure 4. However, the extent of this improvement depends not only on the amount of reinforcement added but also on the effectiveness of its dispersion within the matrix. The nanocomposite showed a noticeable improvement in hardness with increasing reinforcement content, reaching a maximum value of 31.9 HV at 2 wt.% Al_2_O_3_ at 4 h ultrasonication, consistent with previous studies [67,68,69]. This enhancement can be attributed to the uniform dispersion of Al_2_O_3_ particles within the aluminum matrix, which hinders dislocation motion through the Orowan strengthening mechanism and improves the mechanical performance compared to the base alloy [70]. Furthermore, the addition of nanoparticles promotes grain refinement and enhances interfacial bonding, consistent with the Hall–Petch effect, leading to further improvements in hardness and compressive strength of the nanocomposites [29,71]. Although the numerical differences in hardness values are relatively small, a consistent increasing trend is observed from 1 wt.% to 2 wt.% Al_2_O_3_ with increasing ultrasonication time. The statistical analysis based on the signal-to-noise ratio (larger-is-better) for hardness shows that the optimal parameters correspond to 2 wt.% Al_2_O_3_ and 4 h ultrasonication. This behavior confirms the relationship between Al_2_O_3_ content, ultrasonication time, and hardness. Beyond 2 wt.% Al_2_O_3_ level, excessive reinforcement leads to nanoparticle agglomeration and non-uniform dispersion. These agglomerates act as defects and stress concentrators, reducing the effectiveness of reinforcement and resulting in a decline in hardness.

**Figure 5:**
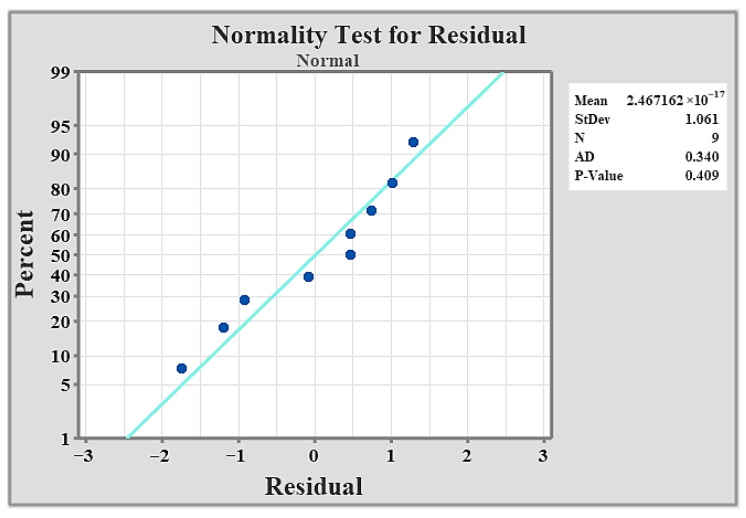
Normality test.

As Figure 5 indicates, the data align closely with the fitted normal distribution line. Moreover, the *p*-value exceeds the 0.05 significance level, confirming that the normality assumption is satisfied. The circles (dots) represent the individual residual data points obtained from the model. Each point indicates the deviation of an observed value from its corresponding predicted value. The straight diagonal line represents the theoretical normal distribution. If the residuals follow a normal distribution, the points are expected to lie along this line. Therefore, when the circles closely align with the diagonal line, it suggests that the residuals are approximately normally distributed, supporting the normality assumption of the statistical.

**Figure 6:**
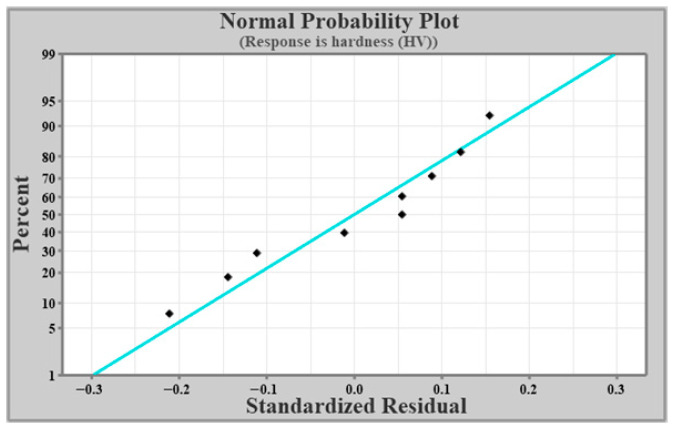
Normal probability for hardness.

Figure 6 presents the normal probability plot for hardness, where most of the data points lie close to the zero standardized residual line. The squares (data points) represent the standardized residuals from the experimental or predicted data. Each point shows how an individual observation deviates from the model’s expected value after standardization. The straight line is the reference normal line, which represents the ideal case where the residuals follow a perfect normal distribution. When the squares lie close to the straight line, it indicates that the residuals are approximately normally distributed, confirming that the model assumptions are satisfied.

**Figure 7:**
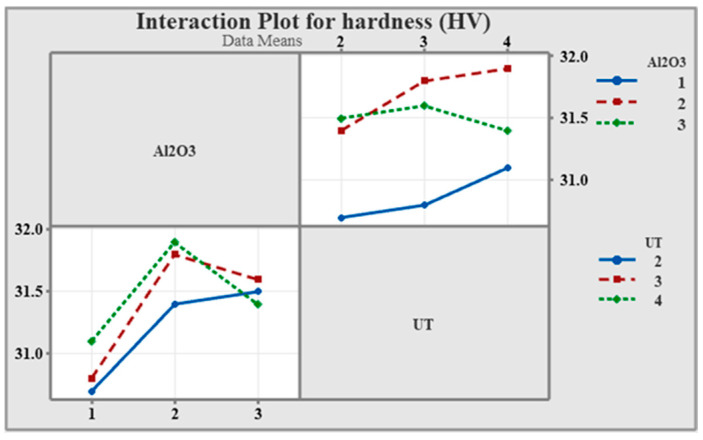
Interaction plot.

As shown in Figure 7, the non-parallel and intersecting lines confirm that hardness is influenced by the combined effect of Al_2_O_3_ content and UT. This suggests that optimal hardness requires careful selection of both parameters together rather than considering each factor independently.

### 3.4. Compressive Strength of AA6082/Al_2_O_3_ Naocomposites

This section presents and discusses the experimental results from compressive strength tests performed on ultrasonicated AA6082/Al_2_O_3_ nanocomposites. The objective is to evaluate the complex effects of UT and Al_2_O_3_ content on the composite’s compressive load-bearing capacity. The unreinforced AA6082 alloy was used as a control sample, showing compressive strength of 154.22 MPa.

#### Effect of Al_2_O_3_ Volume Fraction and Ultrasonication Time on Compressive Strength

**Figure 8:**
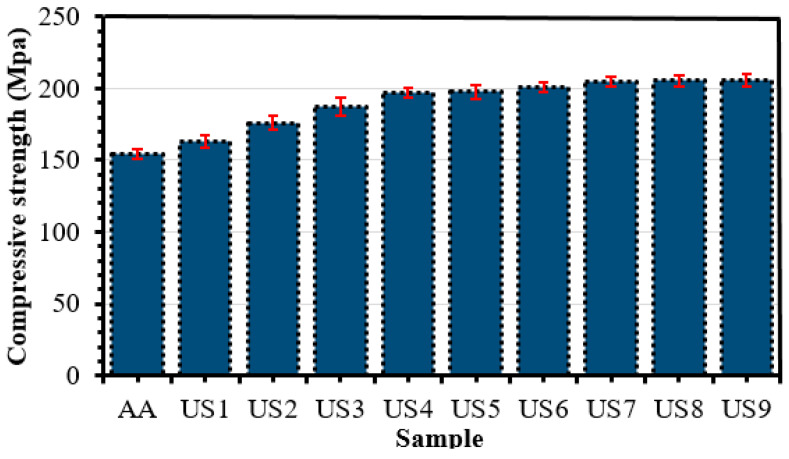
Compressive strength of nanocomposites.

The incorporation of Al_2_O_3_ nanoparticles typically improves the compressive strength of the AA6082 matrix. However, the extent of enhancement is strongly influenced by both reinforcement concentration and UT, as shown in Figure 8. The compressive strength of the composite increased with the addition of Al_2_O_3_ nanoparticles to the base alloy (AA6082). This improvement can be attributed to the effective transfer of load from the aluminum matrix to the Al_2_O_3_ nanoparticles, which was facilitated by nanoparticle uniform distribution within the matrix due to UT. Such behavior indicates strong mechanical bonding between the reinforcement and the matrix. However, the extent of enhancement is strongly influenced by both reinforcement concentration and UT. UT strongly influences the uniform dispersion of reinforcement particles in the matrix, which is crucial for achieving maximum compressive strength, especially at higher nanoparticle contents. In this study, longer ultrasonication durations improved nanoparticle dispersion, reduced agglomeration, and consequently enhanced the compressive strength of the nanocomposites. As illustrated in Figure 8, the compressive strength increases with both Al_2_O_3_ content and ultrasonication time. The compressive strength of 187.22 MPa was recorded at 1 wt.% Al_2_O_3_ with 4 h ultrasonication, similar to [62], while the maximum value of 205.53 MPa was obtained at 3 wt.% Al_2_O_3_ with 4 h ultrasonication, indicating a significant improvement, aligns with the trends reported by [72]. This enhancement can be attributed to improved load transfer from the aluminum matrix to the hard Al_2_O_3_ nanoparticles, as well as grain refinement. These results confirm that increasing reinforcement content and effective ultrasonication contribute to higher compressive strength of the AA6082/Al_2_O_3_ nanocomposites. Although SEM analysis revealed non-uniform distribution and partial agglomeration of Al_2_O_3_ nanoparticles at 3 wt.%, the compressive strength continued to increase. This behavior can be attributed to the load-bearing capacity of the hard ceramic particles and the reduced sensitivity of compressive loading to particle agglomeration. Under compressive stress, agglomerated particles act as rigid load-supporting regions, resulting in an overall improvement in compressive strength.

**Figure 9:**
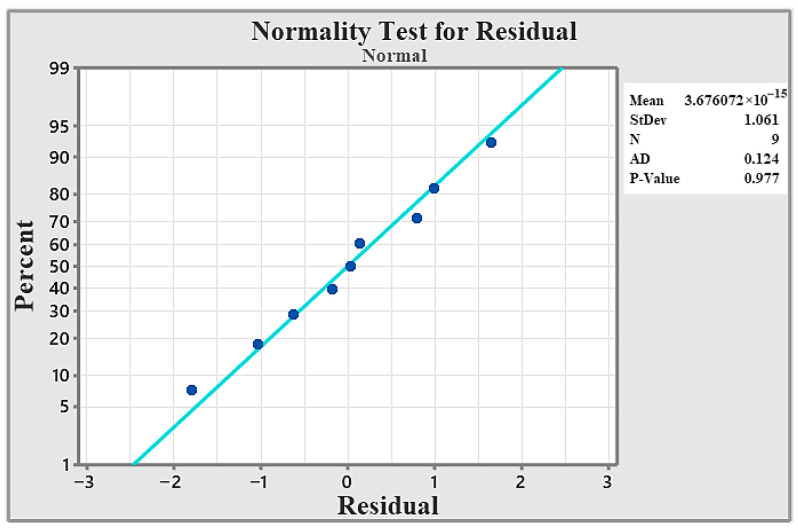
Normality test.

The normality assumption was confirmed using the residual normal probability plot Figure 9. The points closely follow the fitted line, and the Anderson-Darling test produced a *p*-value of 0.977 (>0.05), indicating that the data are normally distributed. The dots represent the individual residual data points obtained from the model, where each point shows the deviation between the observed and predicted values. The straight diagonal line corresponds to the theoretical normal distribution, indicating where the points would fall if the residuals were perfectly normally distributed. When the circles closely follow this line, it suggests that the residuals are approximately normally distributed, thereby supporting the normality assumption of the statistical model.

**Figure 10:**
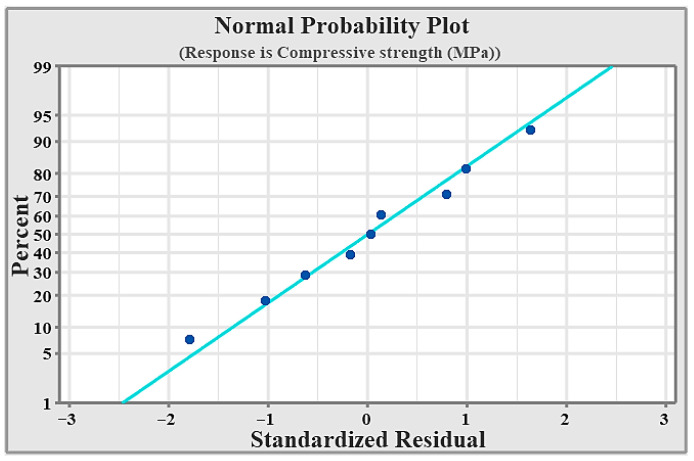
Normal probability for compressive strength.

Figure 10 shows the normal probability plot for compressive strength, where most data points are closely aligned with the zero standardized residual line. The circles represent the standardized residuals from the experimental or predicted data. Each circles shows how an individual observation deviates from the model’s expected value after standardization. The straight line is the reference normal line, which represents the ideal case where the residuals follow a perfect normal distribution. When the squares lie close to the straight line, it indicates that the residuals are approximately normally distributed, confirming that the model assumptions are satisfied.

**Figure 11:**
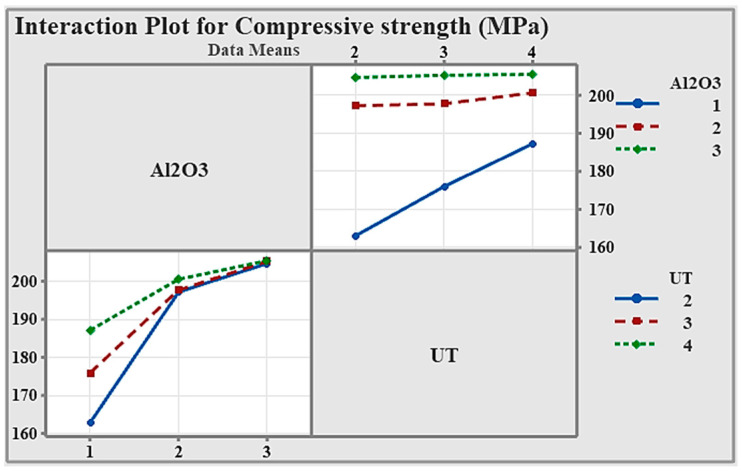
Interaction plot.

The interaction plot in Figure 11 shows that the effect of Al_2_O_3_ concentration on compressive strength depends on the duration of ultrasonication. While the interaction is not statistically strong, the non-parallel trends suggest its changes with reinforcement content.

### 3.5. Optimization

This section focuses on single-response optimization to identify the process parameters that maximize the mechanical properties of aluminum matrix nanocomposites. The Taguchi method incorporates a statistical performance measure known as the signal-to-noise (S/N) ratio, which quantifies how much the observed results deviate from the desired performance. By using the S/N ratio as an objective function, the Taguchi technique helps identify the optimal process parameters that minimize variability and consistently enhance the target performance characteristics. Generally, there are three categories of performance characteristics in the analysis of the S/N ratio. Depending on the performance goal, the S/N ratio is selected from three criteria: larger-is-better, smaller-is-better, or nominal-is-best [73]. In this study, the larger-is-better quality characteristic was selected to evaluate the performance outcomes. The corresponding S/N ratio equations for the larger is better are presented in Equation (1) [74].

(1) $$S/N\;(\mathrm{larger}\;\mathrm{is}\;\mathrm{the}\;\mathrm{better})=-10log\frac{1}{n}\sum_{i=1}^{n}\frac{1}{y^{2}},$$

where n = is the number of experiments, and y = observed response value. The obtained results of mechanical properties were converted into the S/N ratio. The measured mechanical property values were then converted into S/N ratios to analyze the influence of each factor and identify the optimal process conditions.

#### 3.5.1. Analysis of Variance (ANOVA)

Analysis of Variance (ANOVA) is used to determine how strongly different process parameters affect the final results. By measuring each parameter’s contribution to the overall variation in performance, ANOVA identifies which factors have the greatest influence on the process. This helps to identify the most critical parameters for achieving optimal results [75]. A regression equation was developed to predict the mechanical properties of the AMMNCs by analyzing the relationship between the process parameters and the resulting performance outcomes.

#### 3.5.2. Hardness

A consistent assessment of performance is made possible by converting the experimental data into S/N ratios, where a greater ratio denotes better material behavior. Equation (1) was used to compute the S/N ratio. ANOVA was used to further analyze the results and determine how much the control factors, reinforcement content, and UT, had an impact on the nanocomposites’ hardness. In addition, ANOVA was employed to examine the interaction effects between these processing parameters. All statistical analyses were performed at a significance level of 5% (0.05), corresponding to a 95% confidence level. The experimental results, associated S/N ratios, and Taguchi-predicted values are presented in Table 4, while the response table for S/N ratios is provided in Table 5. The ANOVA results are summarized in Table 6.

Table 5 specifically presents the response table for S/N ratios, which ranks the control factors according to their influence on hardness. This ranking is based on the delta value, representing the range of S/N ratio variation attributed to each factor. The results indicate that the Al_2_O_3_ reinforcement content has the most significant effect on hardness, followed by UT.

As shown in Table 6, the percentage contribution analysis reveals the relative influence of each control factor on hardness. The Al_2_O_3_ reinforcement content was identified as the most significant factor, contributing 82.25% (Sequential sum of square (Seq SS value of 0.087671)) to the observed variation in hardness. This dominant contribution is attributed to the inherently high hardness of Al_2_O_3_ nanoparticles and their ability to promote grain refinement, which together significantly enhance the composite’s hardness. UT was the second most influential factor, contributing 8.33% (Seq SS value of 0.008882). Its effect is primarily associated with promoting uniform dispersion of the reinforcement within the matrix, which is critical for achieving improved and consistent hardness across the nanocomposite. These findings clearly demonstrate the critical roles of Al_2_O_3_ reinforcement content and UT in optimizing the mechanical properties of the nanocomposites. The residual error accounts for only 9.42% of the total variation, demonstrating that the developed model is within an acceptable range. The goodness of fit is further confirmed by the high coefficient of determination (R^2^ = 90.58%) and adjusted R^2^ value (R^2^ (adj) = 81.17%), indicating that the selected control factors explain the vast majority of the observed variation in the response. Statistical analysis shows that Al_2_O_3_ reinforcement has a significant influence on the results (*p* < 0.05), while the effect of UT is not statistically significant.

**Figure 12:**
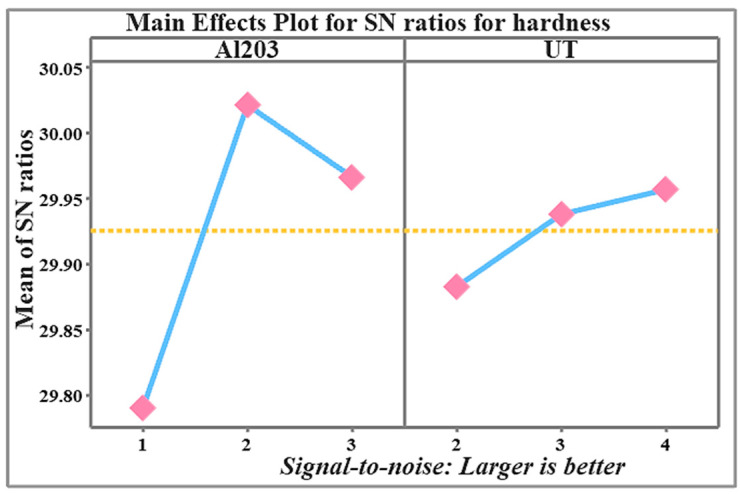
Main effects plot for S/N ratio for hardness of nanocomposite at various process parameters.

The results from Table 4 are graphically represented in Figure 12, which shows the main effect plots for the signal-to-noise ratios of hardness. These plots illustrate the impact of each control factor, specifically, the volume fraction of Al_2_O_3_ nanoparticles and UT, on the response variable. Based on this analysis, the optimal combination of control factor levels was identified as 2 wt.% Al_2_O_3_ and 4 h of ultrasonication time (Al_2_O_3_: 2, UT: 4), which corresponds to the moderate nanoparticle content and longest sonication time. This combination resulted in the maximum hardness values for the nanocomposites, while maintaining process stability and precision. Each square represents the mean signal-to-ratio (S/N) at a particular level of the factor. They show the average hardness response obtained for that level. The blue line connects the mean S/N ratios across the levels of each factor. They show the trend or effect of changing the factor level on hardness. The yellow dotted line is the overall mean S/N ratio across all experiments (grand mean). It serves as a reference line to determine whether a factor level is better (above the line) or worse (below the line) than the overall average.

**Figure 13:**
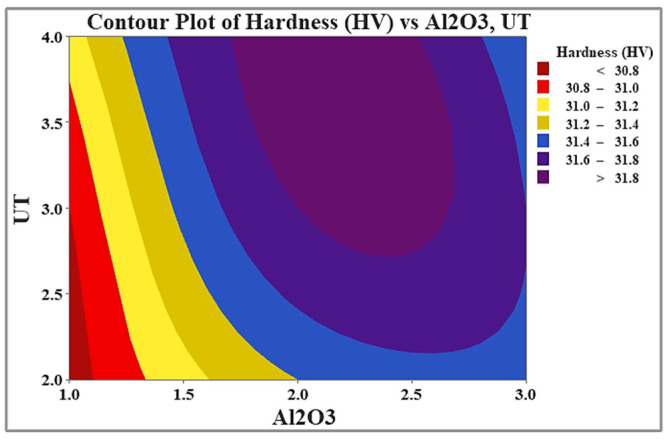
Dependence of hardness on Al_2_O_3_ content and UT.

The experimental hardness results are effectively illustrated using the color-coded maps shown in Figure 13. In these maps, the varying shades visually represent the relationship between hardness and the process parameters. The figure clearly highlights the dependence of hardness on both Al_2_O_3_ content and UT. The maps clearly show that increasing both the Al_2_O_3_ content and the application of UT enhances the hardness of the composite. This trend confirms the beneficial effect of these parameters on hardness, up to an optimal reinforcement level.

#### 3.5.3. Compressive Strength

Consistent interpretation is made possible by converting the experimental results into S/N ratios, where a large S/N ratio denotes superior performance. The S/N ratio was computed using Equation (1). The impact of ultrasonication duration and reinforcing content on the nanocomposites’ compressive strength was assessed using ANOVA. A 5% significance level (*p* = 0.05), which corresponds to a 95% confidence level, was used for all statistical analyses. The experimental compressive strength of the nanocomposites, along with the corresponding S/N ratios and Taguchi-predicted values, are presented in Table 7. The response table for S/N ratios is given in Table 8. The ANOVA results are summarized in Table 9.

Table 8 presents the S/N ratio response table, ranking the control factors based on their influence on compressive strength. The ranking, determined by delta values, shows that Al_2_O_3_ reinforcement content has the greatest effect, followed by UT.

As shown in Table 9, the percentage contribution analysis reveals the relative influence of each control factor on compressive strength. The Al_2_O_3_ reinforcement content was identified as the most significant factor, contributing 81.08% (Sequential sum of square (Seq SS value of 3.1590)) to the observed variation in compressive strength. This dominance indicates that Al_2_O_3_ plays a crucial role in determining the compressive strength of nanocomposites directly. This dominant contribution is attributed to the inherently high hardness of Al_2_O_3_ nanoparticles and their ability to promote grain refinement, which together significantly enhance the composite’s compressive strength. UT was the second most influential factor, contributing 8.20% (Seq SS value of 0.3195). The UT effect is primarily associated with promoting uniform dispersion of the reinforcement within the matrix, which is critical for improving and maintaining consistent compressive strength across the nanocomposite. These findings clearly demonstrate the critical roles of Al_2_O_3_ reinforcement content and UT in optimizing the mechanical properties of the nanocomposites. The residual error accounts for 10.73% of the total variation, demonstrating that the model needs enhancement.

The goodness of fit is further confirmed by the high coefficient of determination (R^2^ = 89.27%) and adjusted R^2^ value (R^2^ (adj) = 78.55%), indicating that the selected control factors explain the vast majority of the observed variation in the response. Statistical analysis shows that Al_2_O_3_ reinforcement has a significant influence on the results (*p* < 0.05), while the effect of UT is not statistically significant.

**Figure 14:**
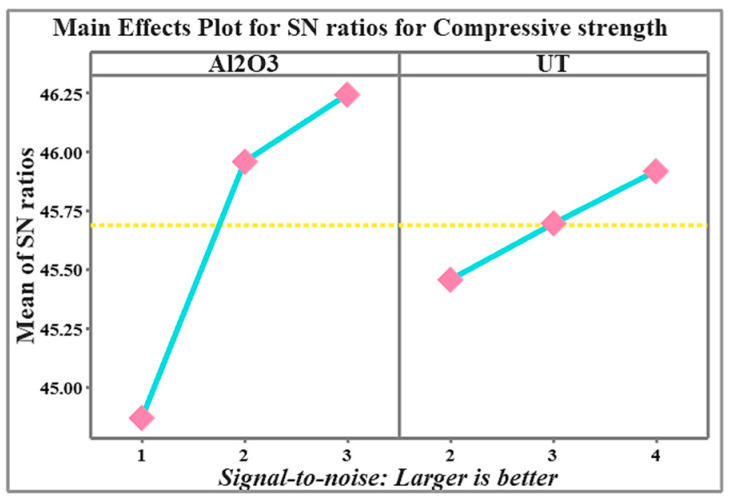
Main effects plot for S/N ratio for compressive strength of nanocomposite at various process parameters.

Figure 14, which displays the major effect plots for the signal-to-noise (S/N) ratios of compressive strength, graphically depicts the data from Table 7. These plots illustrate the impact of each control factor, specifically, the volume fraction of Al_2_O_3_ nanoparticles and UT, on the response variable. Based on this analysis, the optimal combination of control factor levels was identified as 3 wt.% Al_2_O_3_ and 4 h of ultrasonication time (Al_2_O_3_: 3, UT: 4), which corresponds to the highest nanoparticle content and longest sonication time. This combination resulted in the maximum compressive strength values for the nanocomposites, while maintaining process stability and precision. Each square represents the mean signal-to-ratio (S/N) at a particular level of the factor. They show the average compressive strength response obtained for that level. The blue line connects the mean S/N ratios across the levels of each factor. They show the trend or effect of changing the factor level on compressive strength. The yellow dotted line is the overall mean S/N ratio across all experiments (grand mean). It serves as a reference line to determine whether a factor level is better (above the line) or worse (below the line) than the overall average.

**Figure 15:**
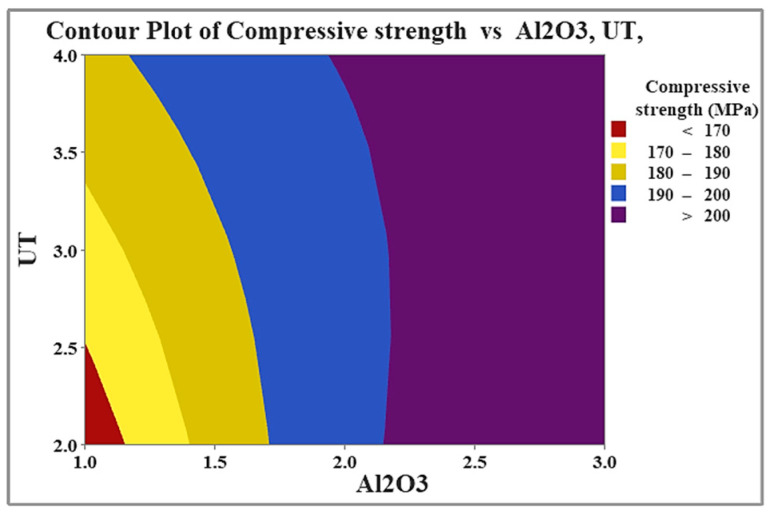
Dependence of compressive strength on Al_2_O_3_ content and UT.

The color-coded maps in Figure 15 provide a good illustration of the experimental compressive strength data. The link between compressive strength and process factors is shown graphically in these maps by the different hues. The figure makes it evident how Al_2_O_3_ content and UT affect compressive strength. The maps clearly show that raising the Al_2_O_3_ volume fraction and UT results in greater compressive strength values, indicating that these parameters have a beneficial impact on the hardness of the composite.

#### 3.5.4. Validation of Results

**Figure 16:**
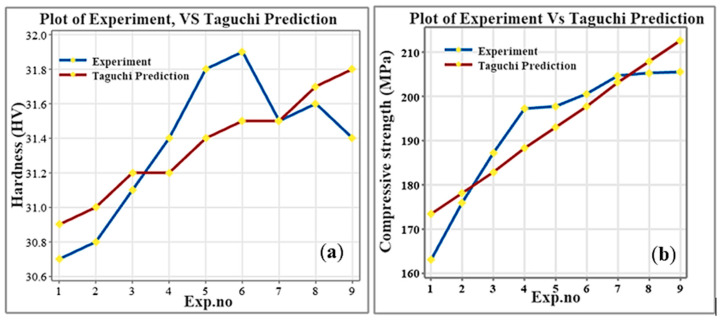
Comparison of experimental and Taguchi-predicted values for (**a**) hardness (HV) and (**b**) compressive strength (MPa).

The regression equation of hardness is HV = 30.322 + 0.317 * Al_2_O_3_ + 0.133 * UT and Regression equation of compressive strength (MPa) = 149.1 + 14.88 * Al_2_O_3_ + 4.73 * UT. The average relative error of 2.64% for compressive strength indicates that the predicted values are very close to the experimental results, showing good reliability and precision. For hardness, the lower error of 0.71% reflects excellent agreement between predicted and measured values. Since both errors are below 5%, the model demonstrates high overall accuracy, with particularly strong performance in predicting hardness. Figure 16a,b presents a comparison between the experimental and predicted values for both properties.

## 4. Limitations of the Study

This study highlights the need for further investigation into advanced dispersion techniques to address the limitations of conventional ultrasonication, particularly at higher nanoparticle reinforcement levels. Although the results confirm that ultrasonication effectively improves dispersion and mechanical properties up to 2 wt.% Al_2_O_3_, its efficiency decreases at higher loadings due to particle agglomeration. In addition, validation of the developed regression models was limited to the available experimental dataset, and broader validation beyond the training data was not conducted. The ultrasonication power was restricted to a maximum of 75 W, and the influence of different solvent systems on nanoparticle dispersion was not examined. Moreover, long-term mechanical stability and wear behavior were not extensively evaluated. Future research should explore higher ultrasonication power, alternative solvent systems, and advanced powder metallurgy techniques to further improve nanoparticle dispersion. Expanding experimental validation, incorporating cross-validation, and assessing long-term mechanical performance would provide a deeper understanding of the material behavior and support further enhancement of nanocomposite properties.

## 5. Conclusions

This study addressed the significant research challenge of assessing how the uniform distribution of nanoparticle reinforcement within a metal matrix impacts the mechanical performance of AMMNCs.

- This research specifically examined the role of ultrasonication duration and volume fraction of Al_2_O_3_ nanoparticle on the microstructural and mechanical properties of AA6082-based nanocomposites by the ultrasonication dispersion method.
- The findings clearly indicated that both UT and nanoparticle concentration play a critical role in optimizing the composite’s performance.
- The study effectively optimized processing parameters for AA6082/Al_2_O_3_ nanocomposites using the Taguchi method, identifying Al_2_O_3_ content as the most influential factor on hardness and compressive strength, with UT as the next significant factor. The optimum hardness was achieved at 2 wt.% Al_2_O_3_ with 4 h of UT, reaching a maximum hardness of 31.9 HV, which represents a 6.33% improvement over the base alloy. Compressive strength, on the other hand, peaked at 3 wt.% Al_2_O_3_ with the same UT duration, reaching 205.53 MPa, a 33% increase compared to the base aluminum alloy.
- Microstructural analysis confirmed relatively uniform Al_2_O_3_ dispersion up to 2 wt.% Al_2_O_3_, contributing to enhanced mechanical properties, while XRD confirmed the presence of Al_2_O_3_ within the aluminum matrix. At 3 wt.% Al_2_O_3_, the microstructure shows non-uniformity and particle agglomeration, which limits further improvements in hardness. Additional microstructural parameters, such as grain size, detailed Al_2_O_3_ distribution, and processing defects, would provide deeper insight into material behavior. However, this study relied on SEM and XRD for phase identification and qualitative assessment of reinforcement dispersion. Advanced analyses, including grain size measurement, EDS mapping, and defect quantification, were not performed due to facility and resource limitations. Nevertheless, the mechanical properties were interpreted using established strengthening mechanisms supported by the available microstructural evidence. These limitations are acknowledged, and detailed analyses are recommended for future work to further improve understanding of AA6082/Al_2_O_3_ nanocomposites. In contrast, compressive strength continues to rise beyond this point due to more effective load transfer between the matrix and reinforcement, highlighting that the mechanisms controlling hardness and compressive strength are different.
- Experimental validation showed minimal deviations, with average relative errors of ≤0.71% for hardness and ≤2.64% for compressive strength, confirming the high accuracy of the optimization approach. The findings highlight strong potential for developing lightweight, high-strength aluminum-based nanocomposites for automotive applications through cost-effective process optimization.
- Validation was not performed in this study due to time and cost limitations. Future work will focus on strengthening statistical reliability through cross-validation; additional experimental verification through analyses, including grain size measurement, EDS mapping, and defect quantification; alternative optimization methods; and assessment of long-term mechanical stability and wear behavior. Further improvements may be achieved by using higher ultrasonication power, exploring different solvent systems, and integrating advanced powder metallurgy techniques.
- In conclusion, the study demonstrates that ultrasonication effectively improves nanoparticle dispersion up to an optimal Al_2_O_3_ content; however, beyond 2 wt.% Al_2_O_3_, show signs of non-uniform distribution and agglomeration occur, indicating that more advanced fabrication methods are required to maintain uniform dispersion and desirable mechanical performance. This research contributes meaningfully to the existing literature by highlighting the critical interplay between reinforcement content and ultrasonication duration in the development of high-performance AA6082-based nanocomposites.

## Figures and Tables

**Table 1:** Chemical composition of aluminum alloy (AA6082).

Si	Fe	Cu	Mn	Mg	Cr	Zn	Ti	Al
0.7–1.2	0.388	0.086	0.4	0.6–1.2	0.1	0.06	0.1	other

**Table 2:** Control factors and their levels.

S.NO	Input Parameters	Symbol	Unit	Levels		
				L1	L2	L3
1	Al_2_O_3_	Al_2_O_3_	wt.%	1	2	3
2	Ultrasonication time	UT	Hour	2	3	4

**Table 3:** Experimental design using L9 orthogonal array.

Sample	Al_2_O_3_	UT
US1	1	2
US2	1	3
US3	1	4
US4	2	2
US5	2	3
US6	2	4
US7	3	2
US8	3	3
US9	3	4

**Table 4:** Experimental results using L9 orthogonal array.

Sample	Hardness (HV)	S/N for Hardness (dB)	Prediction for Hardness (dB)
US1	30.7	29.7428	29.7228
US2	30.8	29.7710	29.7868
US3	31.1	29.8552	29.8594
US4	31.4	29.9386	29.9541
US5	31.8	30.0485	30.0181
US6	31.9	30.0758	30.0908
US7	31.5	29.9662	29.9231
US8	31.6	29.9937	29.9783
US9	31.4	29.9386	29.9971

**Table 5:** Response table for signal-to-noise ratios (larger-is-better) for hardness.

Level	Al_2_O_3_	UT
1	29.79	29.88
2	30.02 *	29.94
3	29.97	29.96 *
Delta	0.23	0.07
Rank	1	2

The * denotes the optimal values determined from Signal-to-Noise (S/N) ratio analysis using the Taguchi method, representing the best-performing factor level combination instead of raw experimental measurements.

**Table 6:** Analysis of variance for signal-to-noise ratio (larger-is-better) of hardness.

**Source**	**DF**	**Seq SS**	**Adj SS**	**Adj MS**	**F-Value**	***p*-Value**	**Contribution**
Al_2_O_3_	2	0.087671	0.087671	0.043835	17.47	0.011	82.25%
UT	2	0.008882	0.008882	0.004441	1.77	0.281	8.33%
Residual Error	4	0.010036	0.010036	0.002509			9.42%
Total	8	0.106589					100.00%
R-Sq (90.58%), R-Sq (adj) (81.17%)							

DF: degree of freedom, Seq SS: sequential sum of square, Adj SS: adjusted sum square, and Adj MS: adjusted mean square.

**Table 7:** Experimental results using L9 orthogonal array.

Sample	Compressive Strength (MPa)	S/N for Compressive Strength (dB)	Prediction for Compressive Strength (dB)
US1	163.04	44.2459	44.6346
US2	175.99	44.9098	44.8721
US3	187.22	45.4470	45.0960
US4	197.24	45.8999	45.7240
US5	197.77	45.9232	45.9616
US6	200.63	46.0479	46.1855
US7	204.70	46.2224	46.0096
US8	205.30	46.2478	46.2471
US9	205.53	46.2575	46.4710

**Table 8:** Response table for signal-to-noise ratios (larger-is-better) for compressive strength.

Level	Al_2_O_3_	UT
1	44.87	45.46
2	45.96	45.69
3	46.24 *	45.92 *
Delta	1.37	0.46
Rank	1	2

* Denotes the optimal values determined from Signal-to-Noise (S/N) ratio analysis using the Taguchi method, representing the best-performing factor level combination instead of raw experimental measurements.

**Table 9:** Analysis of variance for signal-to-noise ratio (larger-is-better) of compressive strength.

**Source**	**DF**	**Seq SS**	**Adj SS**	**Adj MS**	**F-Value**	***p*-Value**	**Contribution**
Al_2_O_3_	2	3.1590	3.1590	1.5795	15.12	0.014	81.08%
UT	2	0.3195	0.3195	0.1597	1.53	0.321	8.20%
Residual Error	4	0.4179	0.4179	0.1045			10.73%
Total	8	3.8964					100.00%
R-Sq (89.27%), R-Sq (adj) (78.55%)							

DF: degree of freedom, Seq SS: sequential sum of square, Adj SS: adjusted sum square, and Adj MS: adjusted mean square.

## Data Availability

The original contributions presented in this study are included in the article. Further inquiries can be directed to the corresponding authors.
